# Supplementary material for: Metastatic organotropism in small cell lung cancer
Source: bioRxiv. 2025 Jan 24:2024.10.07.617066. Originally published 2024 Oct 8. Preprint. [Version 2] doi: 10.1101/2024.10.07.617066 (PMC11483079; doi:10.1101/2024.10.07.617066)
Supplement: Supplement 1 [file media-1.pdf]

Supplementary Information: Metastatic organotropism in small cell lung cancer

Manan Krishnamurthy[1,2], Anjali Dhall[1#], Sarthak Sahoo[3#], Christopher W. Schultz[1], Michelle A. Baird[4], Parth Desai[1,5], Jacob Odell[6,7], Nobuyuki Takahashi[1,8], Michael Nirula[1], Sophie Zhuang[1], Yue Huang[1], Brett Schroeder[1], Yang Zhang[1], Maria Sebastian Thomas[1], Christophe Redon[1], Christina Robinson[9], Lai Thang[9], Lilia Ileva[10], Nimit L. Patel[10], Joseph D. Kalen[10], Alice-Anaïs Varlet[6], Noam Zuela-Sopilniak[7], Ankita Jha[4], Darawalee Wangsa[11], Donna Butcher[12], Tamara Morgan[12], Alyah N. Afzal[13], Raj Chari[13], Karim Baktiar[11], Suresh Kumar[1], Lorinc Pongor[1,14], Simone Difilippantonio[9], Mirit I. Aladjem[1], Yves Pommier[1], Mohit Kumar Jolly[3], Jan Lammerding[7], Ajit Kumar Sharma[1], and Anish Thomas[1*]

[#] Equal contribution

[1]Developmental Therapeutics Branch, National Cancer Institute, National Institutes of Health, Bethesda, MD
[2]Medical Scientist Training Program, Icahn School of Medicine at Mount Sinai, New York, NY, 10029, USA
[3]Department of Bioengineering, Indian Institute of Science, Bangalore, India
[4]Cell and Developmental Biology Center, National Heart, Lung and Blood Institute, National Institutes of Health; Bethesda, USA
[5]Department of Hematology & Medical Oncology, Fox Chase Cancer Center, Philadelphia, PA
[6]Weill Institute for Cell and Molecular Biology, Cornell University, Ithaca, NY 14853, USA; Graduate Field of Biochemistry, Molecular and Cell Biology, Cornell University, Ithaca, NY 14853, USA.
[7]Weill Institute for Cell and Molecular Biology, Cornell University, Ithaca, NY 14853, USA; Meinig School of Biomedical Engineering, Cornell University, Ithaca, NY 14853, USA.
[8]Department of Medical Oncology, National Cancer Center East Hospital, Kashiwa, Japan
[9]Animal Research Technical Support, Laboratory Animal Sciences Program, Leidos Biomedical Research, Inc., Frederick National Laboratory for Cancer Research, NIH, Frederick, MD 21701
[10]Small Animal Imaging Program, Laboratory Animal Sciences Program, Leidos Biomedical Research, Inc., Frederick National Laboratory for Cancer Research, Frederick, MD 21701, USA
[11]Genetics Branch, Center for Cancer Research, National Cancer Institute, Bethesda, Maryland.
[12]Molecular Histopathology Laboratory, Laboratory of Animal Sciences Program, Frederick National Laboratory for Cancer Research, Frederick, MD, USA.
[13]Laboratory Animal Sciences Program, Genome Modification Core, Frederick National Laboratory for Cancer Research, Frederick, USA
[14]Cancer Genomics and Epigenetics Core Group, Szeged, Hungary

Corresponding Author: Anish Thomas, Center for Cancer Research, National Cancer Institute, Building 10, Room 4-5330, Bethesda, MD 20892; Ph: 240-760-7343; Fax: 954-827-0184; Email: anish.thomas@nih.gov

Grant information: This study was supported by the Center for Cancer Research, the Intramural Program of the NCI (ZIA BC 011793). A.T: grants to NCI from EMD Serono Research & Development, AstraZeneca, Gilead Sciences, and ProLynx. JL: NIH R35 GM153257, NSF URoL-2022048, Volkwagen Foundation A130142

Table S1- Cell-line information

Cell line	Procurement method	Site of metastasis	Generation	Cell line injected to yield	Generation + site name used throughout paper
4-lymph node	Human rapid autopsy	Cervical lymph node	Parental		
5-liver	Human rapid autopsy	Right lobe posterior inferior liver	Parental		
6-liver	Human rapid autopsy	Right lobe inferior most	Parental		Parent
12-lung	Human rapid autopsy		Parental		
18-adrenal	Human rapid autopsy	Left adrenal	Parental		
400L	Mouse necropsy	Liver	1	6-liver	Gen1LMD
404L	Mouse necropsy	Liver	1	6-liver	Gen1LMD
406B	Mouse necropsy	Brain	1	6-liver	Gen1BMD
408B	Mouse necropsy	Brain	1	6-liver	Gen1BMD
431L	Mouse necropsy	Liver	2	406B	Gen2LMD
431B	Mouse necropsy	Brain	2	406B	Gen2BMD
438L	Mouse necropsy	Liver	2	406B	Gen2LMD
438B	Mouse necropsy	Brain	2	406B	Gen2BMD

Table S2- Immunohistochemical staining of SCLC markers

Tumor Sample	Target	Marker Type	Typical [1]	IHC Type	Total Cells	% Positive	H-Score
RA-22-6	Chromogranin A	Traditional NE Marker	(74% cases)	Cytoplasmic	9111	89.5	210.3
RA-22-6	Synaptophysin	Traditional NE Marker	some not all cells	Cytoplasmic	15830	99.1	293.4
RA-22-6	INSM1	NE Marker	92%	Nuclear	74025	87.9	144.4
RA-22-6	CD56	Traditional NE Marker	75-100%(>80% of cases)	Cytoplasmic	27528	99.5	298.0
RA-22-6	Ki-67	Cancer Proliferative activity	60%	Nuclear	25155	58.7	93.2
406-Liver	Chromogranin A	Traditional NE Marker	(74% cases)	Cytoplasmic	5472	81.5	149.4

406-Liver	Synaptophysin	Traditional NE Marker	some not all cells	Cytoplasmic	3033	99.2	296.5
406-Liver	INSM1	NE Marker	92%	Nuclear	3671	74.3	87.6
406-Liver	CD56	Traditional NE Marker	75-100%(>80% of cases)	Cytoplasmic	2201	99.9	299.4
406-Liver	Ki-67	Cancer Proliferative activity	60%	Nuclear	2495	42.4	52.5
406-Brain	Chromogranin A	Traditional NE Marker	(74% cases)	Cytoplasmic	1396	48.7	75.9
406-Brain	Synaptophysin	Traditional NE Marker	some not all cells	Cytoplasmic	1786	99.7	298.7
406-Brain	INSM1	NE Marker	92%	Nuclear	2079	89.2	124.1
406-Brain	CD56	Traditional NE Marker	75-100%(>80% of cases)	Cytoplasmic	1801	99.8	297.6
406-Brain	Ki-67	Cancer Proliferative activity	60%	Nuclear	17530	58.1	86.0

Table S3- Epigenetically linked differentially expressed genes in each cluster

**Upregulated genes**						**Downregulated genes**				
**Cluster 1**	**Cluster 2**	**Cluster 3**	**Cluster 5**	**Cluster 0**		**Cluster 0**				
PPARGC1A	CDH1	C1orf21	SOX3	L3MBTL4	DMKN	SLC35F4	GRIN2B	NRXN3	VAV3	ACVR1C
	ADGRD2	CCDC152	ARHGEF9	MET	YBX3	LINC01194	NPAS3	DHRS2	SLC43A3	LINC00461
	CST4	CACNG4	PKD1L3	NTS	ATP2B3	ADARB2	FRMD4B	LRRTM3	MARCH4	MAPK10
	CD163L1	SAMD13	MYO5B	DSP	TRPM3	LINC01470	ZNF91	TRHDE	RAB27B	
		CTDSPL	COL4A4	TNS3	OPRD1	BCL11B	PREX2	NMUR2	CRIM1	
		RHOJ	COL4A3	PAPPA	PRPH2	SEMA3A	PCDH17	LIN7A	EYA2	
		LINC01151	TGFBR3	SLC18A1	NR2F1	KCNIP4	AIG1	STMN2	COL4A6	
		NRXN2	PTH2R	CNTN1	ARHGAP22	DSCAM	SKAP1	SNTB1	STC1	
		GADL1	CR1L	RASSF9	PKHD1	ZNF536	CTNNA3	RAMP1	NKD1	
		SPAG17		PRSS12	ZNF486	CADM2	CNTNAP4	PPP2R2B	UGT2A1	

		ZBTB7C		LGR6	MIR100HG	GABBR2	GRIK1	ARID5B	LINC00626	
		KCTD16		ZNF90	PDZRN4	AMPH	DISC1FP1	AFF3	RNF152	
				PPP4R4	CHL1	CST1	CLMP	CACNA1E	SLC8A3	
					ERVMER61-1	FGF13	ZEB2	PDE9A	CELF5	

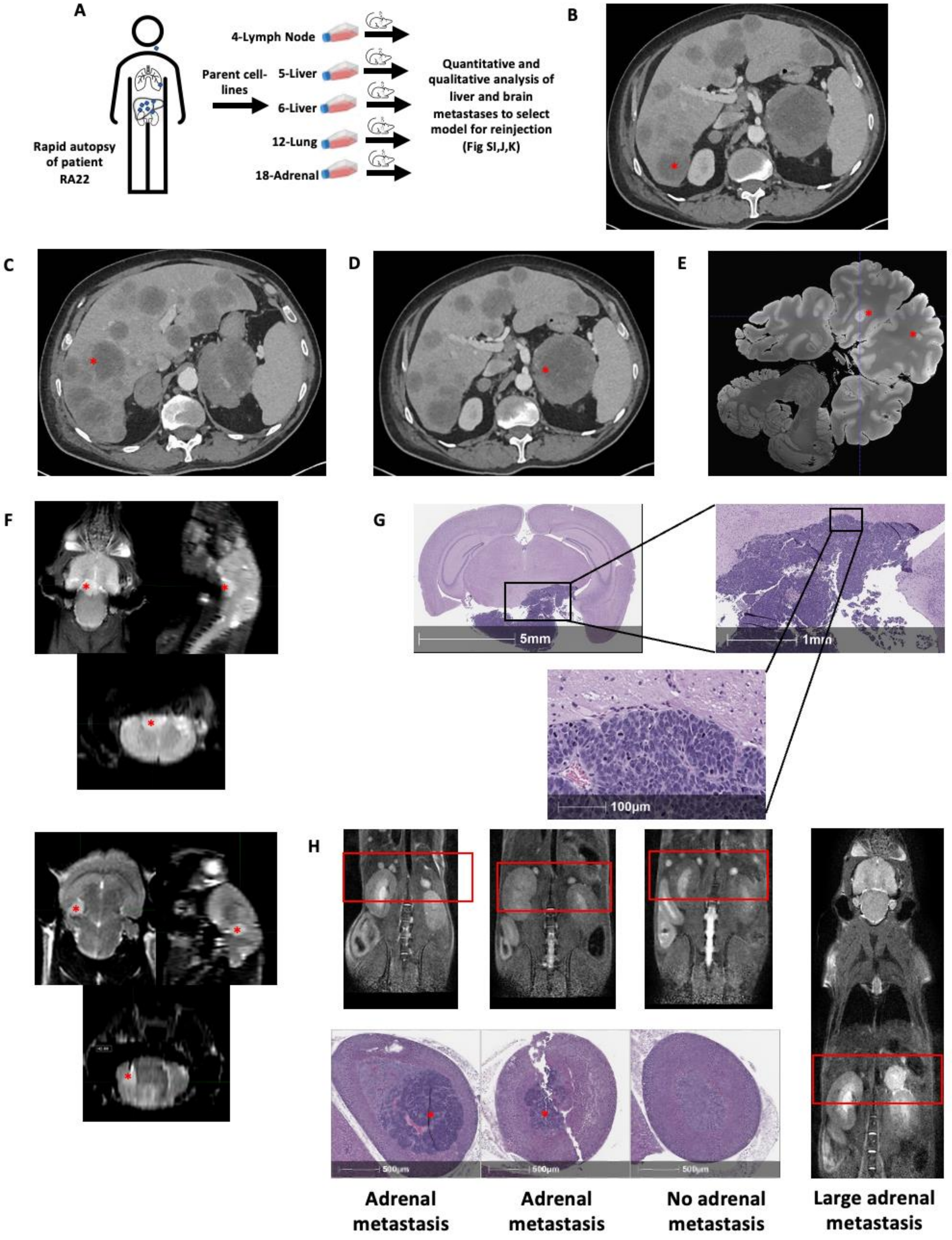

A
Rapid autopsy of patient RA22
Parent cell-lines
4-Lymph Node
5-Liver
6-Liver
12-Lung
18-Adrenal
Quantitative and qualitative analysis of liver and brain metastases to select model for reinjection (Fig SI,J,K)
B
C
D
E
F
G
5mm
1mm
100µm
H
500µm
500µm
500µm
Adrenal metastasis
Adrenal metastasis
No adrenal metastasis
Large adrenal metastasis

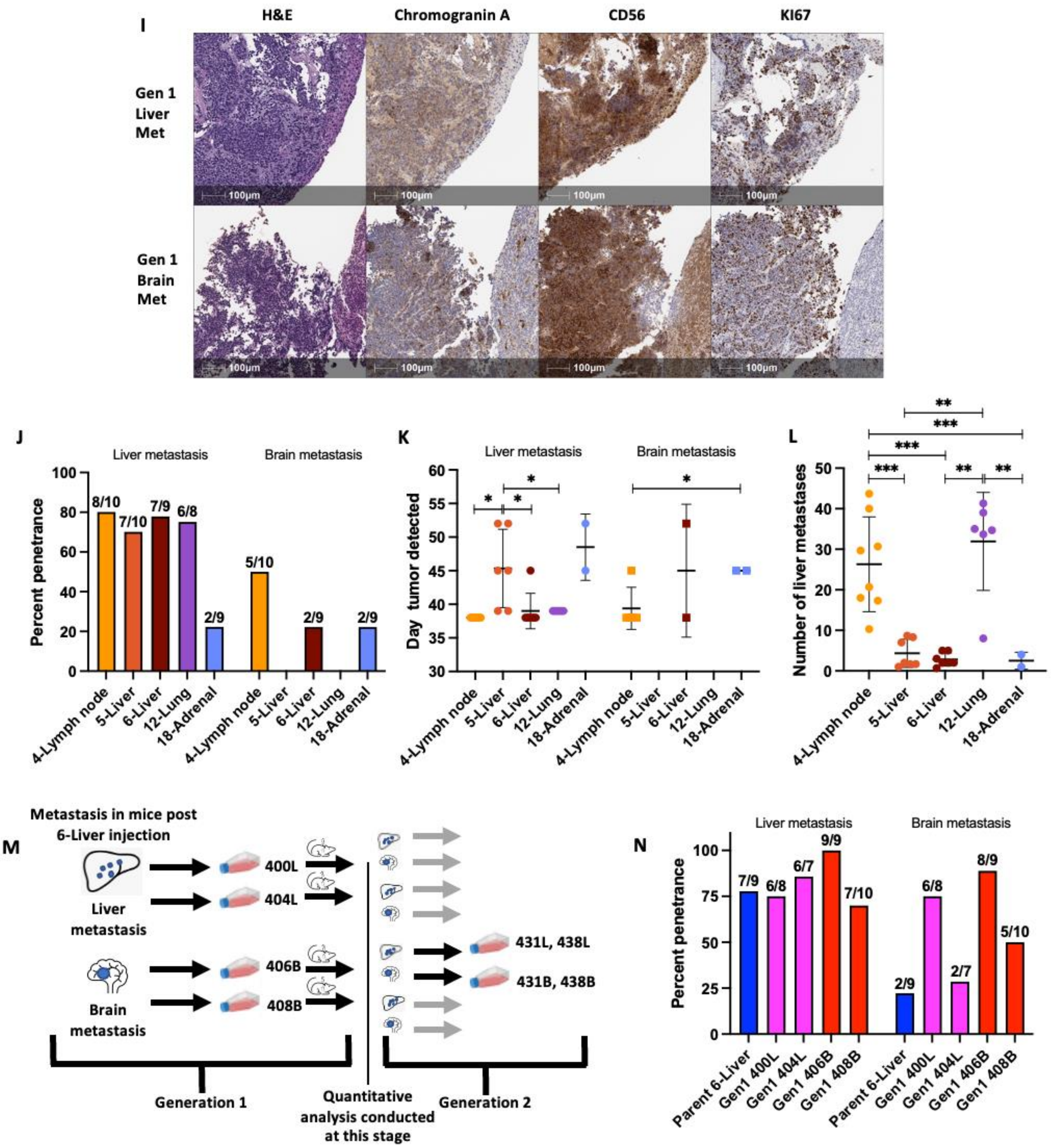

I
H&E
Chromogranin A
CD56
KI67
Gen 1 Liver Met
Gen 1 Brain Met
100µm
J
Liver metastasis
Brain metastasis
Percent penetrance
8/10
7/10
7/9
6/8
2/9
5/10
2/9
2/9
4-Lymph node
5-Liver
6-Liver
12-Lung
18-Adrenal
K
Liver metastasis
Brain metastasis
Day tumor detected
L
Number of liver metastases
M
Metastasis in mice post 6-Liver injection
Liver metastasis
Brain metastasis
400L
404L
406B
408B
431L, 438L
431B, 438B
Generation 1
Quantitative analysis conducted at this stage
Generation 2
N
Liver metastasis
Brain metastasis
Percent penetrance
7/9
6/8
6/7
9/9
7/10
2/9
6/8
2/7
8/9
5/10
Parent 6-Liver
Gen1 400L
Gen1 404L
Gen1 406B
Gen1 408B

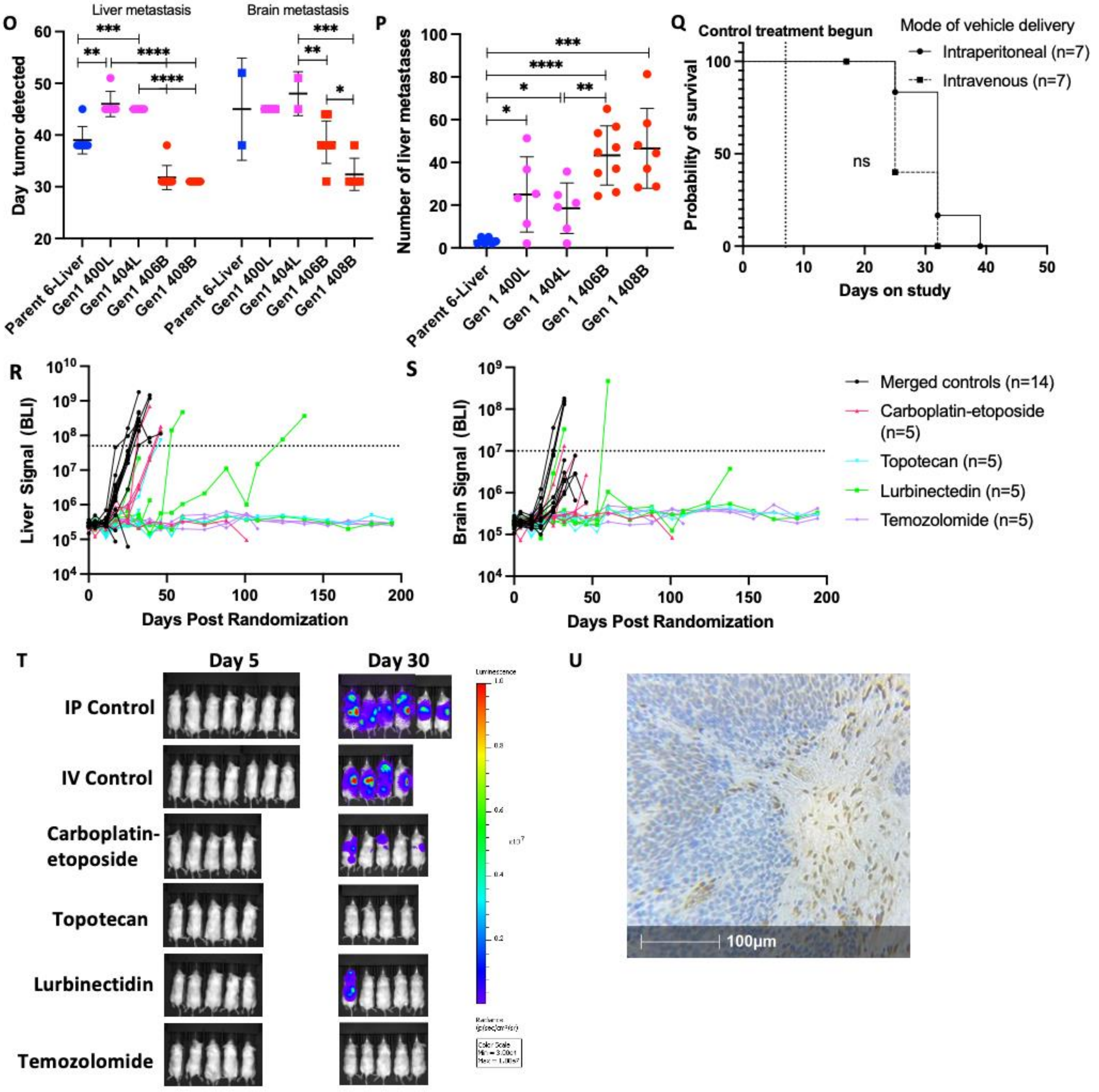

Figure S1: Establishment and characterization of a patient-derived model of SCLC metastases and organ tropism
A: Schema of mouse experiments with five parental cell lines.
B: CT scan for patient RA22 showing right posterior inferior lobe liver metastasis used to make 5-liver cell line, asterisks indicate metastasis
C: CT scan for patient RA22 showing right most inferior lobe liver metastasis used to make 6-liver cell line, asterisks indicate metastasis
D: CT scan for patient RA22 showing left adrenal metastasis used to make 18-adrenal cell line, asterisks indicate metastasis
E: Brain, CT post-mortem, asterisks indicate metastasis
F: Coronal, sagittal, and transverse cuts of typical (top) and rare (bottom) route of brain colonization, MRI, after RA22-4 (lymph node derived) cell line injection
G: Zoom of brain metastasis cells in Fig. 1F
H: Complexity identifying adrenal metastases on MRI
I: H&E and IHC for lung specific, tumor specific, and additional neuroendocrine factors in generation 1 metastases.

J: Percent penetrance (% of mice that formed tumors) of brain and liver metastases, assessed by MRI. RA22-4, RA22-5, RA22-6, RA22-12, RA22-18. Mice excluded if found dead or sacked due to bodyweight loss prior to appearance of first met in cohort. Mice numbers indicated above each bar.
K: Time to develop liver and brain metastasis in days. Mice with no metastases excluded.
L: Number of liver metastases. Mice with no metastases excluded
M: Schema of mouse experiments with five parental cell lines. RNA-seq data collected for generation 1 and 2 cell lines labeled.
N: Percent penetrance (% of mice that formed tumors) of brain and liver metastases, assessed by MRI. RA22-6, 400L, 404L, 406B, 408B. Mice excluded if found dead or sacked due to bodyweight loss prior to appearance of first met in cohort. Mice numbers indicated above each bar.
O: Time to develop liver and brain metastasis in days. Mice with no metastases excluded.
P: Number of liver metastases. Mice with no metastases excluded
Q: Survival (Kaplan-Meyer) curve of mice injected with 406B cells + vehicle. Vehicle delivered by routes indicated.
R: BLI signal from liver. The dotted horizontal line is the threshold for high tumor burden.
S: BLI signal from brain. The dotted horizontal line is the threshold for high tumor burden.
T: Representative image of BLI for each treatment condition
U: MGMT IHC from patient RA22 right most inferior lobe liver metastasis (used to make 6-liver cell line)

All significance indicated is following parametric unpaired T-tests with Welch's correction. *p<0.05, **p<0.01, ***p<0.001, ****p<0.0001. All error bars are mean with standard deviation unless mentioned otherwise. BMD: Brain metastasis derived; LMD cell lines: 400L, 404L; BMD cell lines: 406B, 408B. All patient Computational Topography (CT) transverse slices 2.5 weeks pre-mortem unless otherwise indicated.

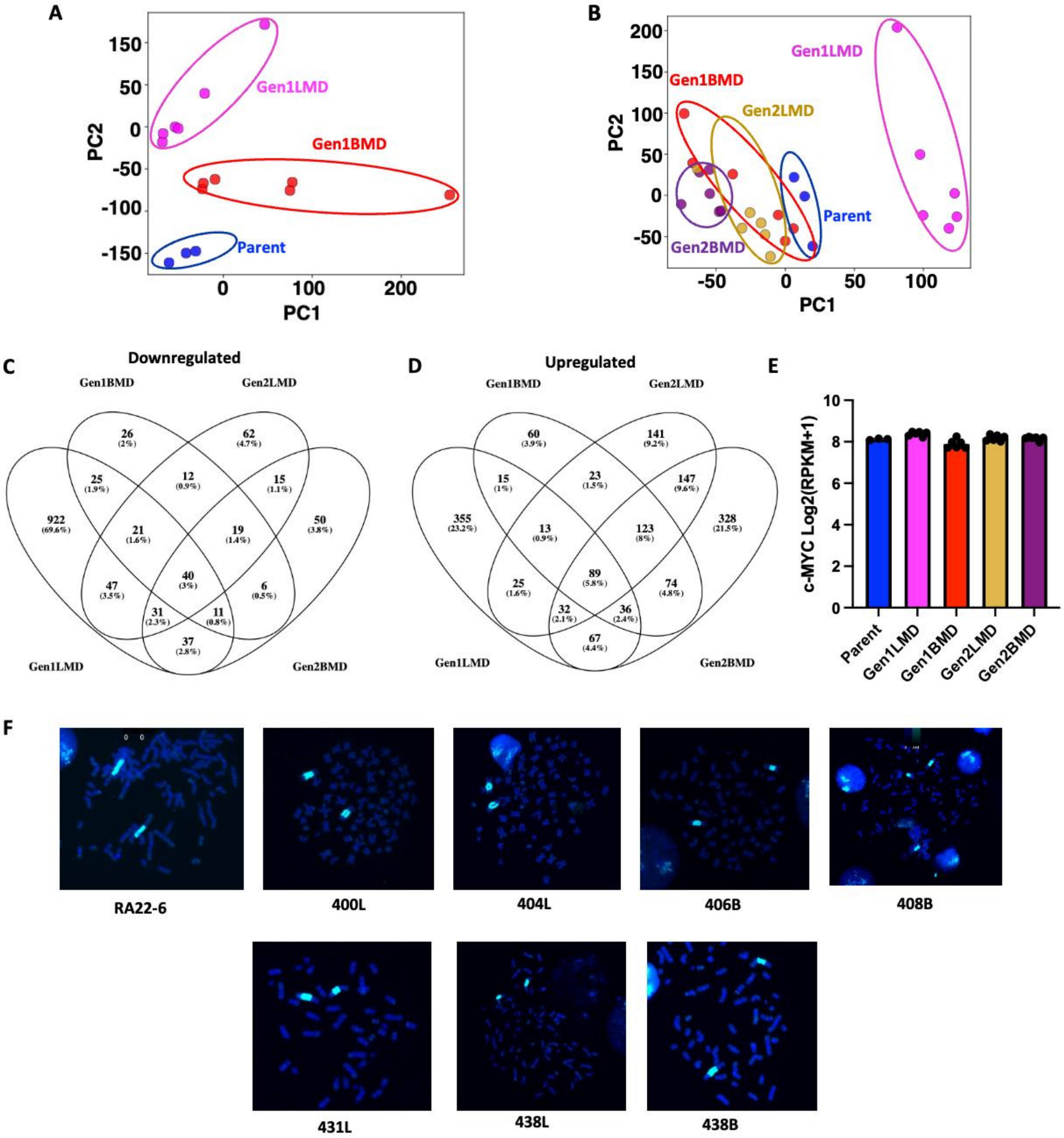

Figure S2: Global gene expression analysis of RA22-6 and derived cell lines
A: PCA of Parent and generation 1 all genes RNA-seq
B: PCA of Parent, generation 1, and generation 2 all genes RNA-seq
C: Venn diagram depicting differentially (Pvalue <0.05 & abs(logFC) >1.5) downregulated genes in Gen1LMD, Gen2LMD, Gen1BMD, Gen2BMD compared to Parent.
D: Venn diagram depicting differentially (Pvalue <0.05 & abs(logFC) >1.5) upregulated genes in Gen1LMD, Gen2LMD, Gen1BMD, Gen2BMD compared to Parent.
E: Gene expression of MYC
F: MYC DNA-FISH for MYC in Parent RA22-6, generation 1, and generation 2 cell lines

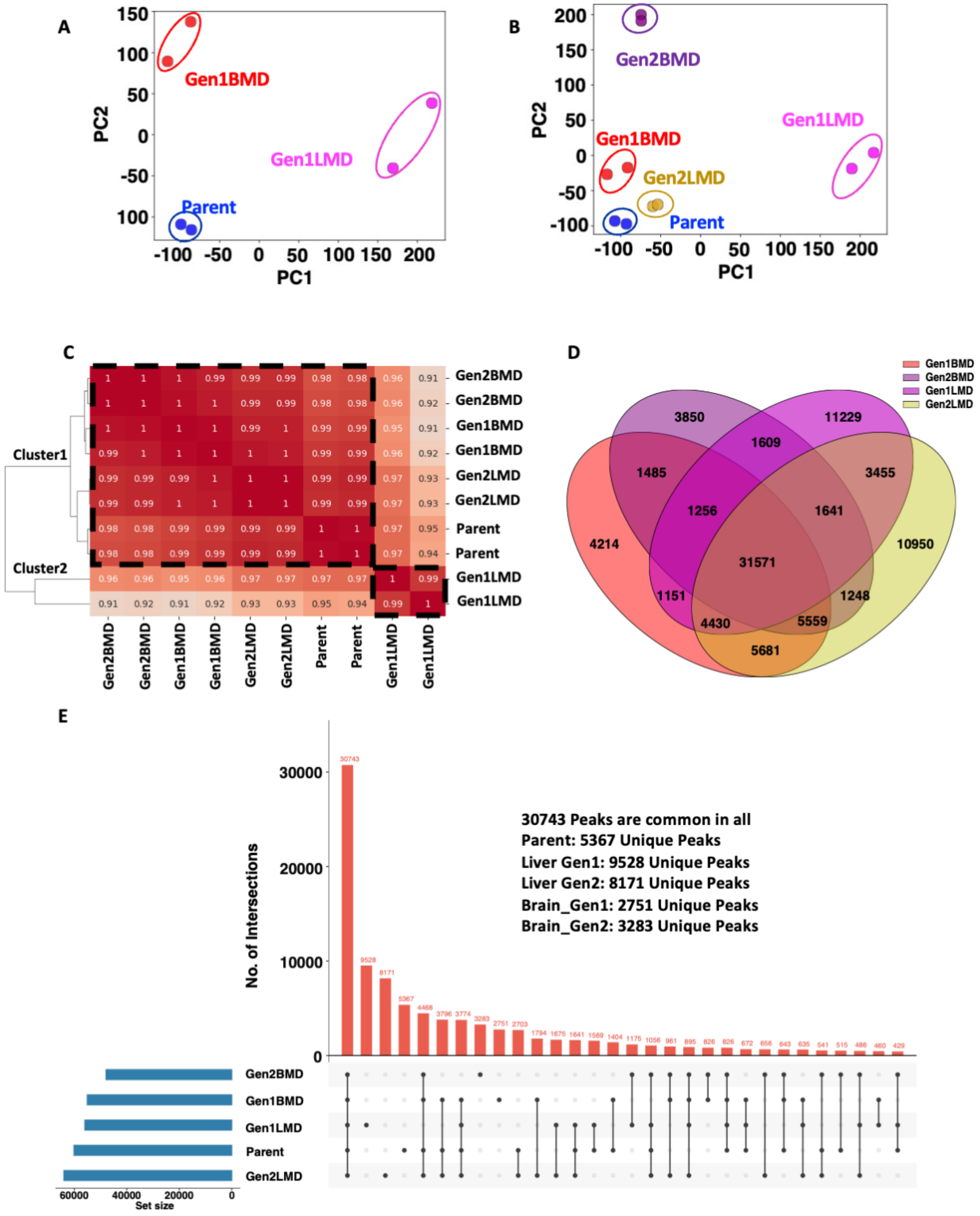

Figure S3: Chromatin accessibility associated with SCLC organ-specific metastatic behaviors
A: PCA of Parent and generation 1 all peaks ATAC-seq

B: PCA of Parent, generation 1, and generation 2 all peaks ATAC-seq
C: Pairwise correlation heatmap of open peaks in Parent, generation 1, and generation 2.
D: Venn Diagram depicting unique peaks between Gen1LMD, Gen1BMD, Gen2LMD and Gen2BMD.
E: Bar graph depicting overlap of peaks between Parent, generation 1, and generation 2 all peaks

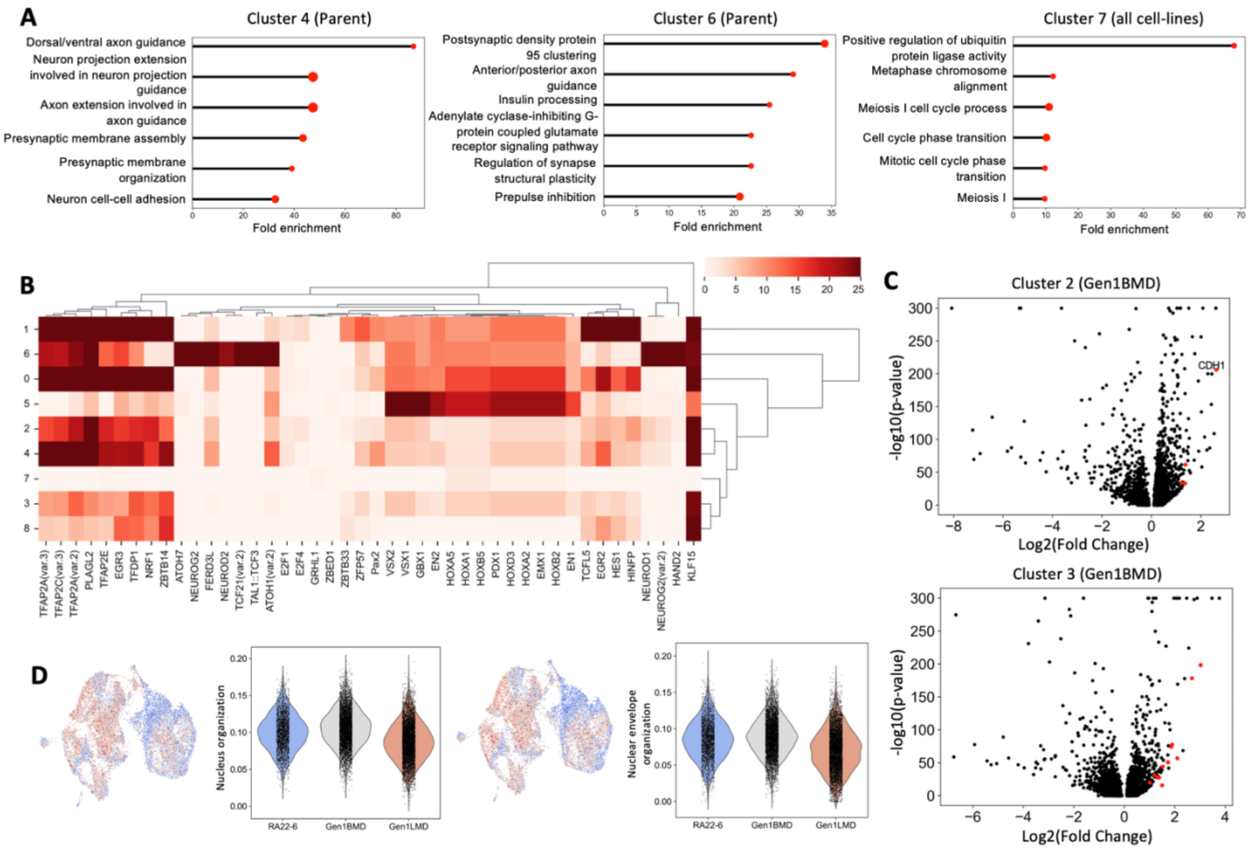

E

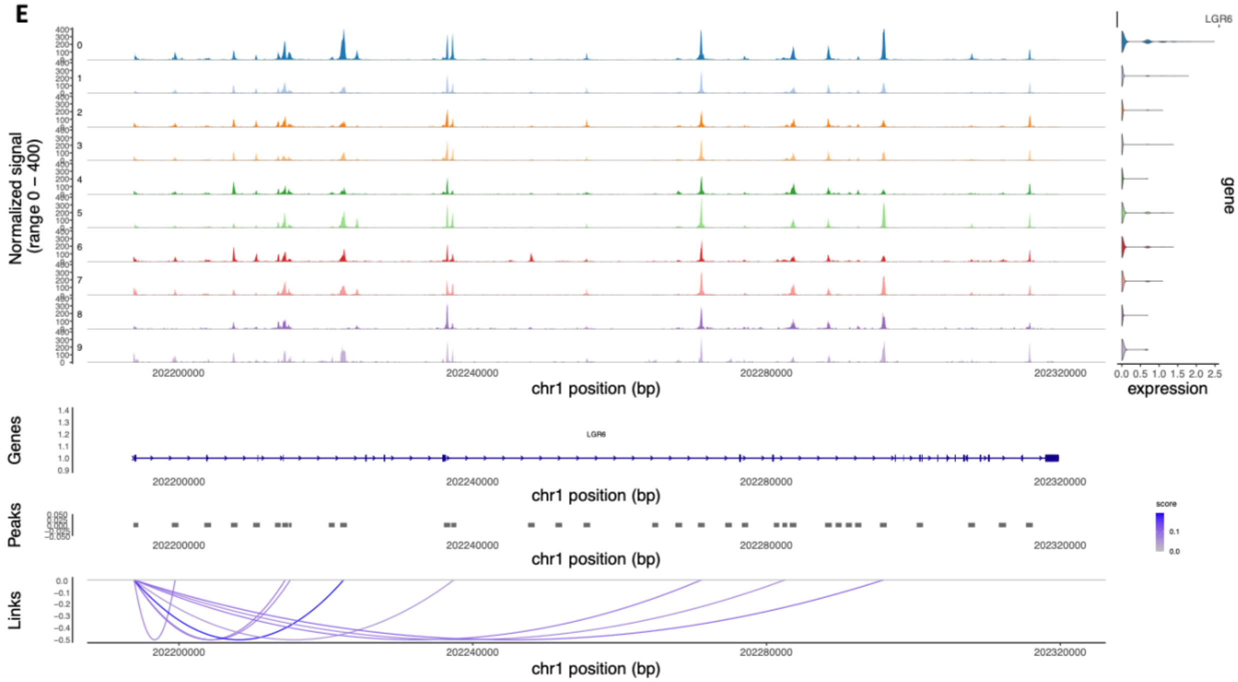

Normalized signal (range 0 – 400)
LGR6
gene
expression
chr1 position (bp)
202200000
202240000
202280000
202320000
Genes
Peaks
score
Links

F

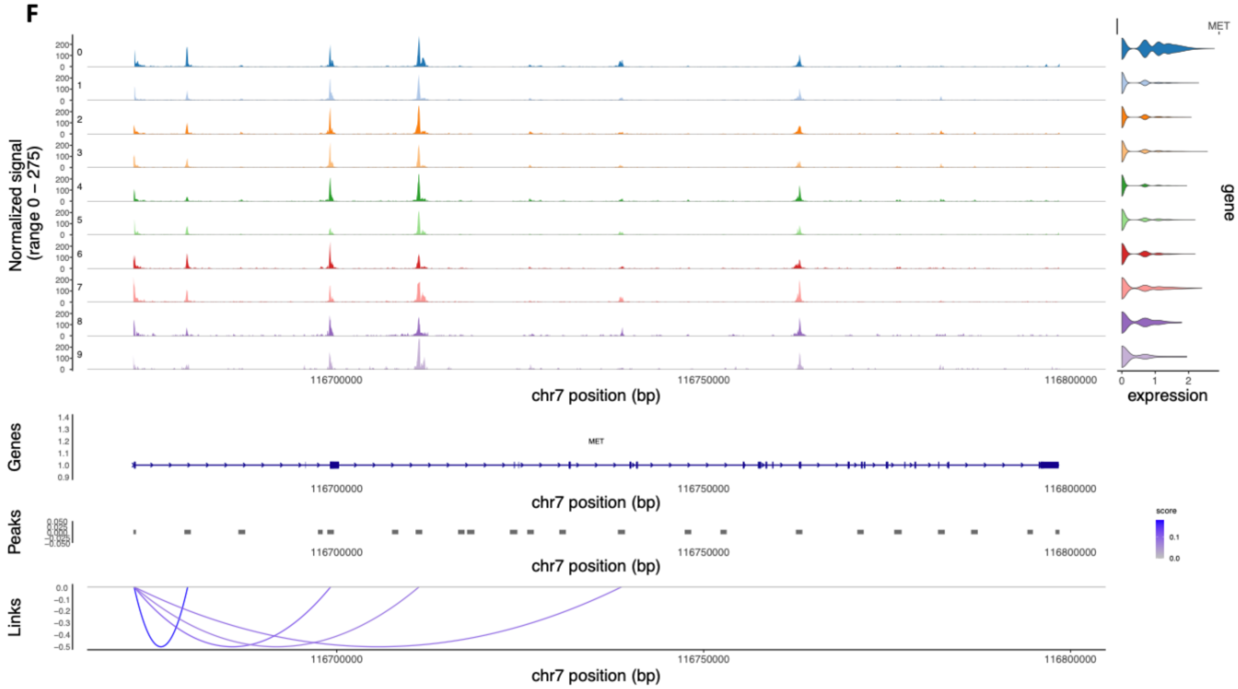

Normalized signal (range 0 – 275)
MET
gene
expression
chr7 position (bp)
116700000
116750000
116800000
Genes
Peaks
score
Links

G

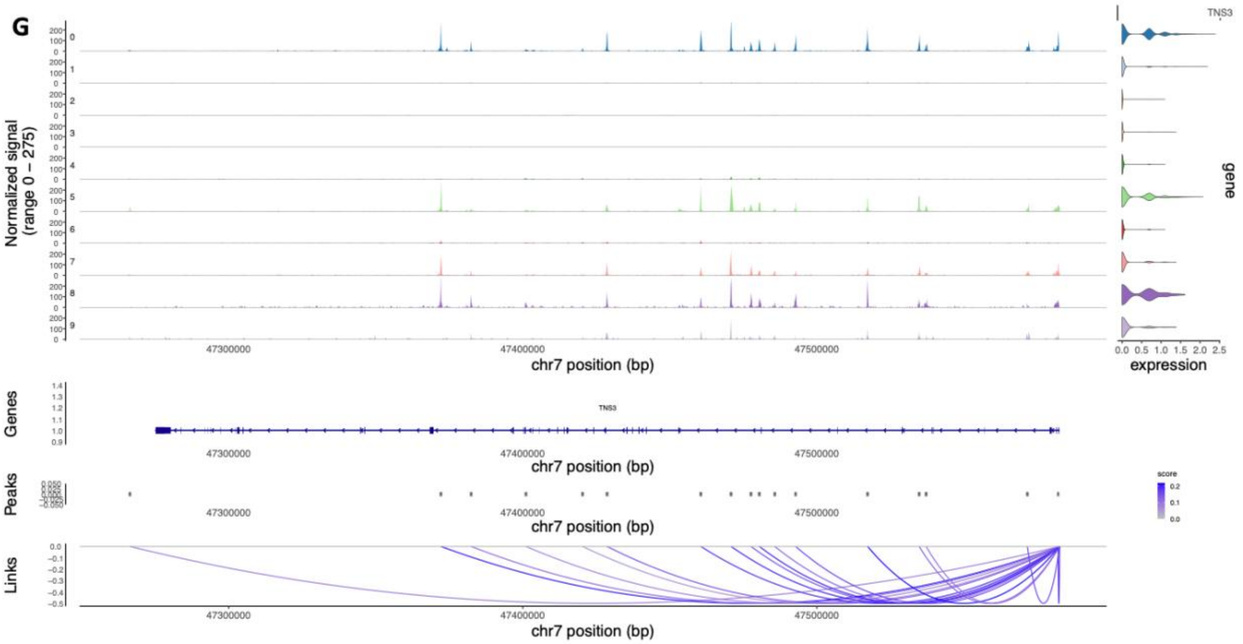
TNS3
Normalized signal (range 0 – 275)
gene
expression
chr7 position (bp)
Genes
Peaks
Links
score
47300000
47400000
47500000

H

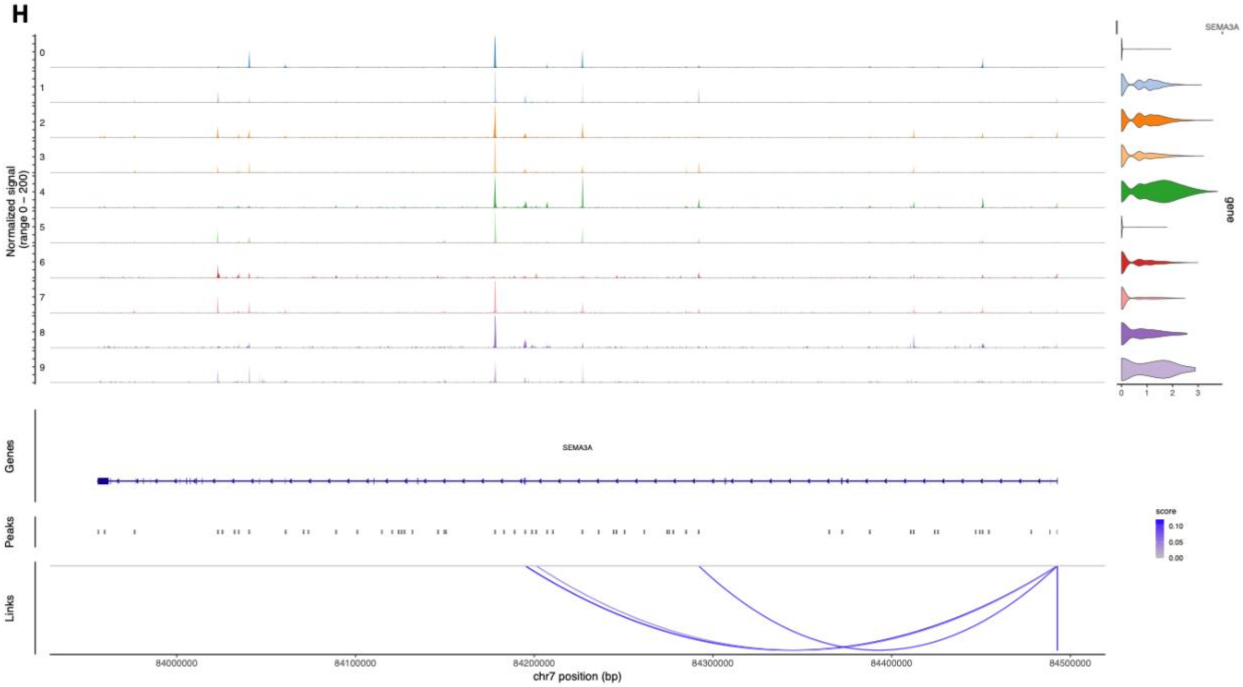
SEMA3A
Normalized signal (range 0 – 200)
gene
Genes
Peaks
Links
score
chr7 position (bp)
84000000
84100000
84200000
84300000
84400000
84500000

I

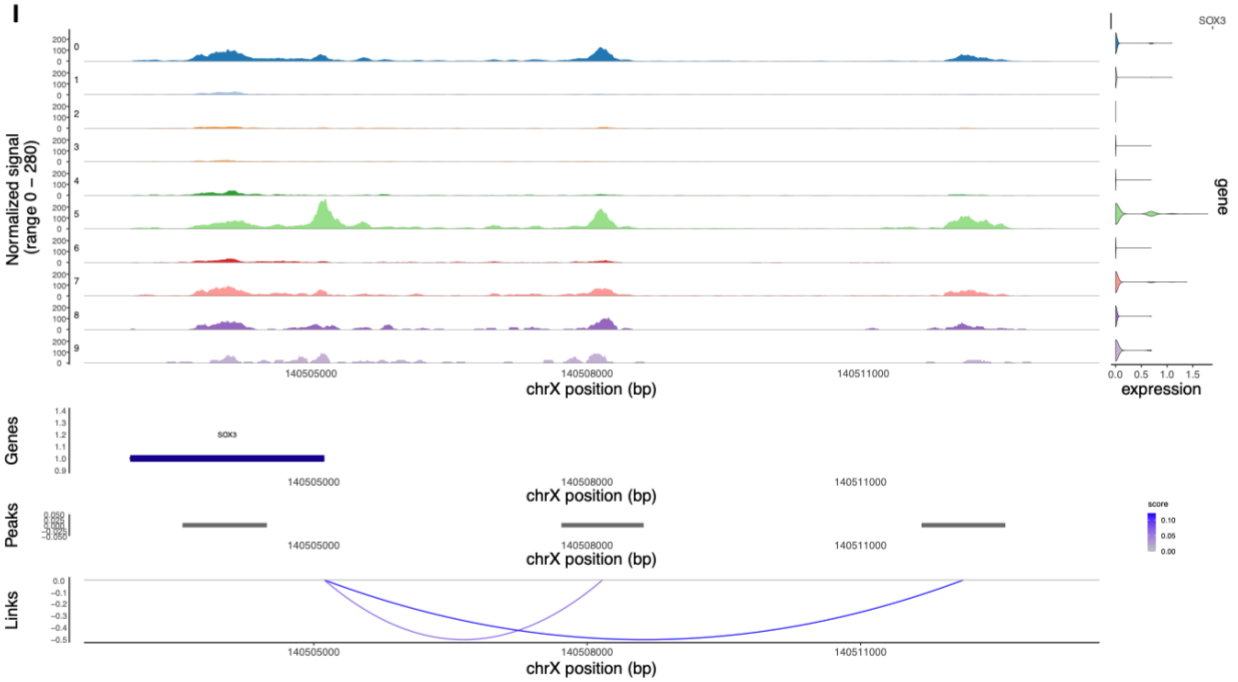
SOX3
Normalized signal
(range 0 – 280)
gene
140505000
140508000
140511000
chrX position (bp)
expression
Genes
SOX3
Peaks
score
0.10
0.05
0.00
Links

J

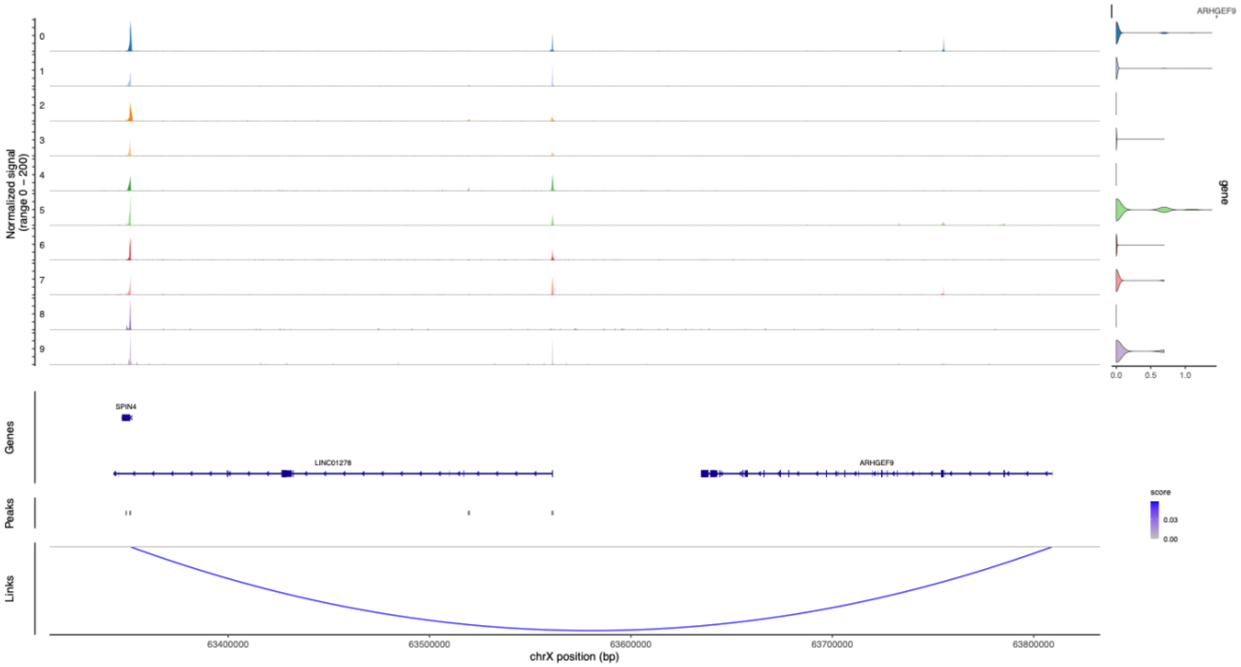
ARHGEF9
Normalized signal
(range 0 – 200)
gene
Genes
SPIN4
LINC01278
ARHGEF9
Peaks
score
0.03
0.00
Links
63400000
63500000
63600000
63700000
63800000
chrX position (bp)

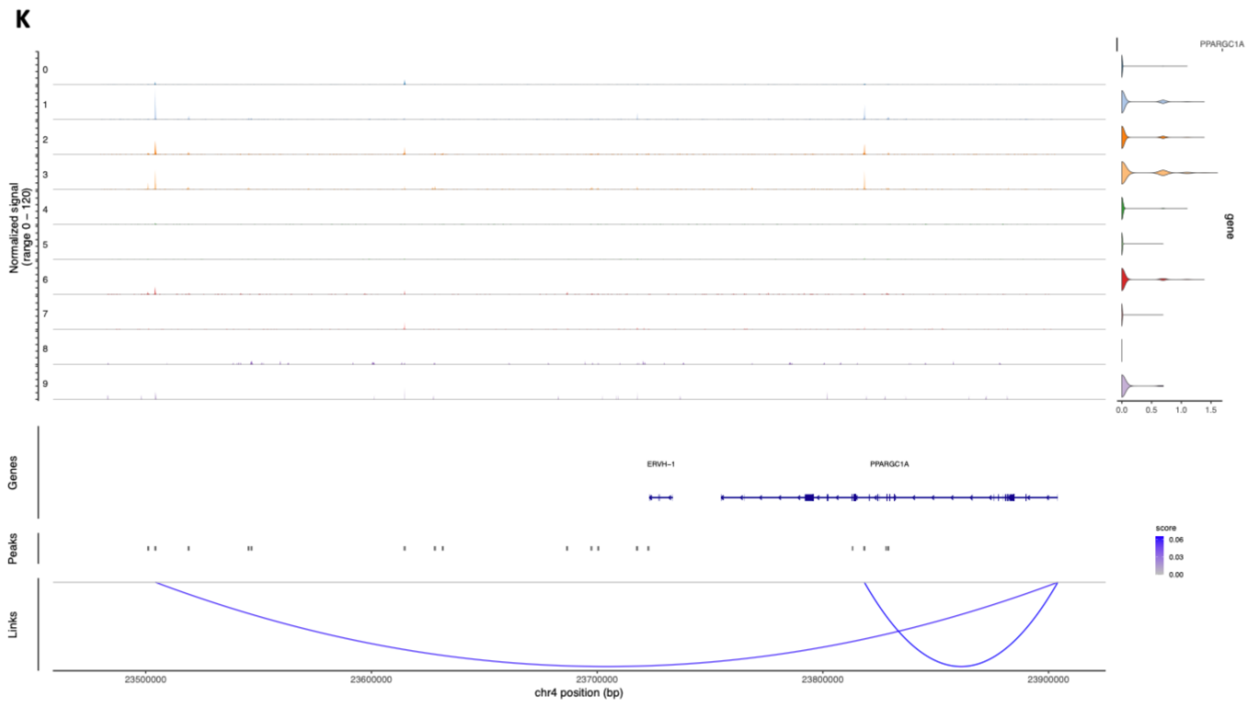

Figure S4: Multiome sequencing supporting data
A: Gene ontology of Parent (Clusters 4.6) and all samples (cluster 7).
B: Top 10 most significant known motifs for all clusters (44 motifs represented on graph as there was overlap). Complete list of motifs in supplemental file.
C: GSEA of nucleosome envelop organization and nucleosome organization from bulk ATAC-seq
D: Volcano plots of differentially expressed genes in individual clusters compared to all others. Red dots indicate genes that also show significant epigenetic linkage.
E: LGR6 coverage plot.
F: MET coverage plot.
G: TNS3 coverage plot.
H: SEMA3A coverage plot.
I: SOX3 coverage plot.
J: ARHGEF9 coverage plot.
K: PPARGC1A coverage plot.

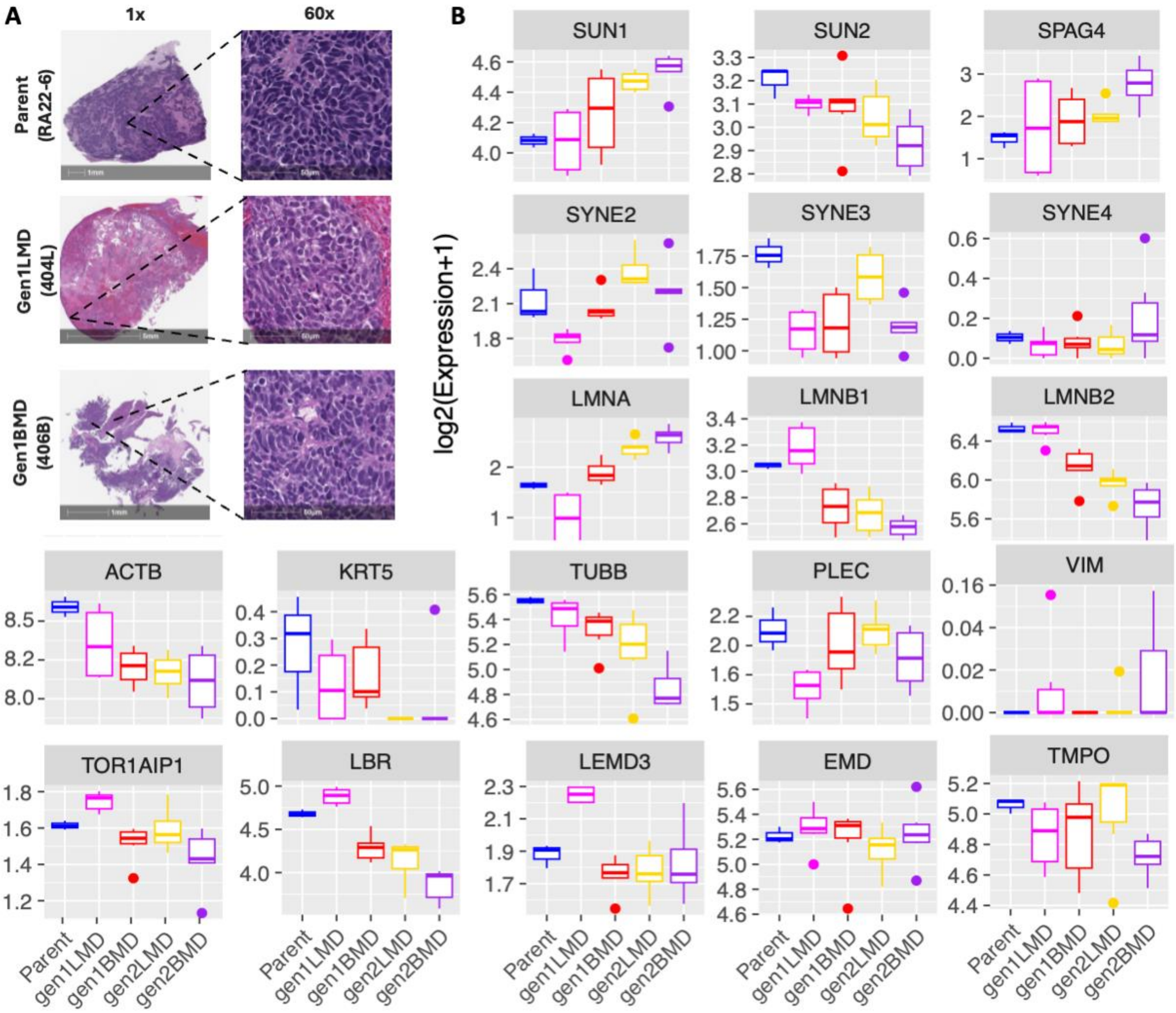

A
1x
60x
Parent (RA22-6)
Gen1LMD (404L)
Gen1BMD (406B)
B
log2(Expression+1)
SUN1
SUN2
SPAG4
SYNE2
SYNE3
SYNE4
LMNA
LMNB1
LMNB2
ACTB
KRT5
TUBB
PLEC
VIM
TOR1AIP1
LBR
LEMD3
EMD
TMPO
Parent
gen1LMD
gen1BMD
gen2LMD
gen2BMD

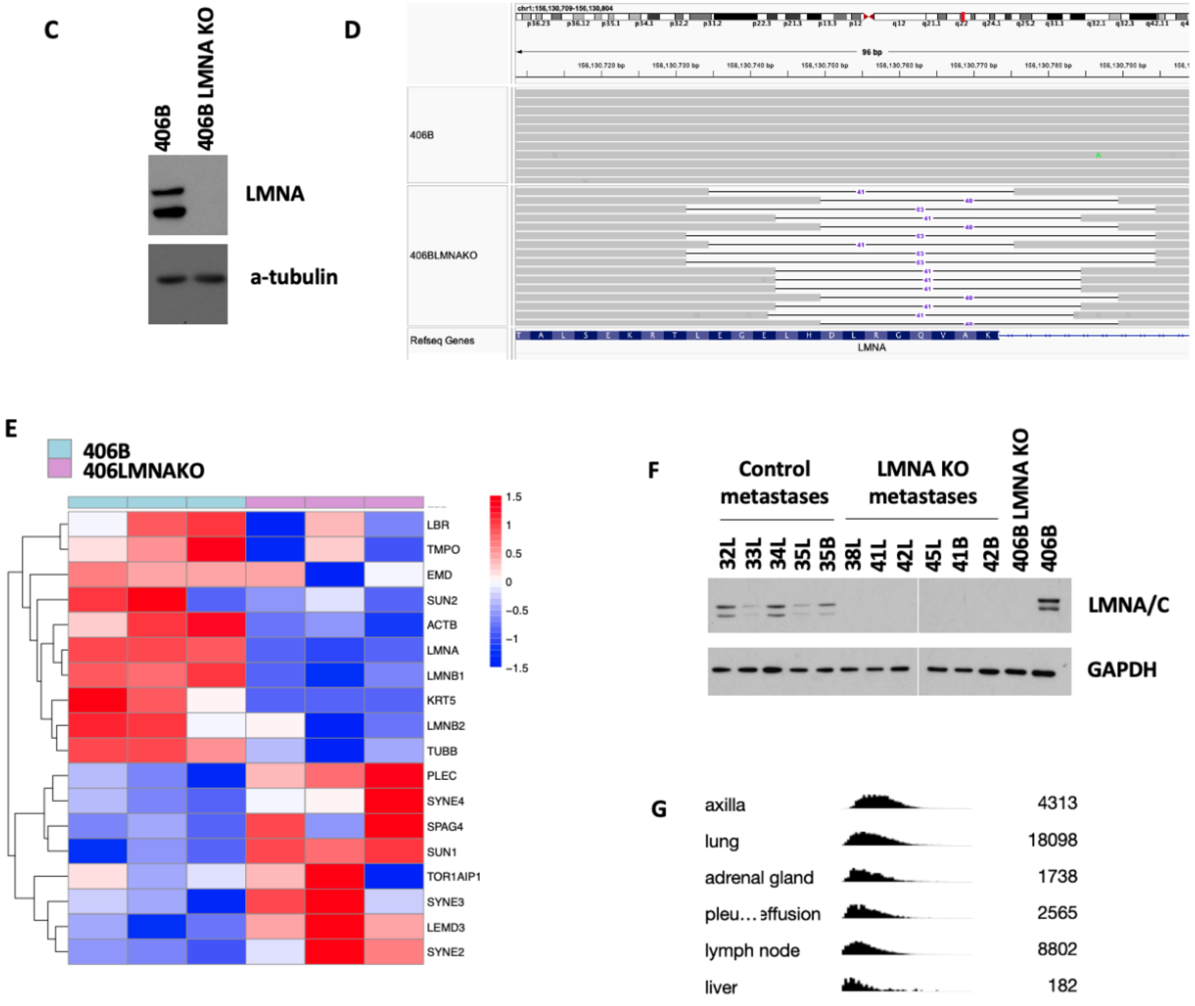

Figure S5: Dysregulation of cellular structure and LMNA KO in SCLC

A: H&E illustrating SCLC nuclei in Parent and generation 1 metastases

B: RNA-seq of LINC complex genes in Parent, generation 1, and generation 2 of SCLC metastasis model (shows progressive dysregulation of LINC)

C: Western blot of LMNA KO in 406B

D: Deep amplicon sequencing to identify mutations in 406B LMNAKO

E: Heatmap of gene expression of LINC complex and related genes in 406B and 406B LMNAKO

F: LMNA/C western blot from metastasis cell lines after in vivo LMNAKO metastasis modeling. Samples were run side-by-side on two different blots (separated by white space above) that were developed at the same time.

G: Distribution of LMNA expression of human SCLC metastatic cells [2] expressing, LMNA stratified by site of metastasis

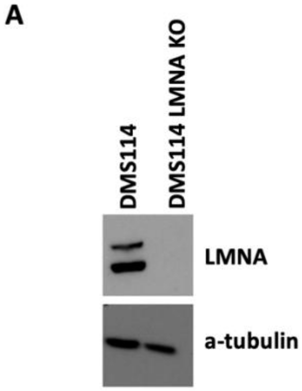

Figure S6: In vitro examination of LMNA role in nuclear deformability, migration, and metastasis
A: Western blot of LMNA KO in DMS114

Movies:

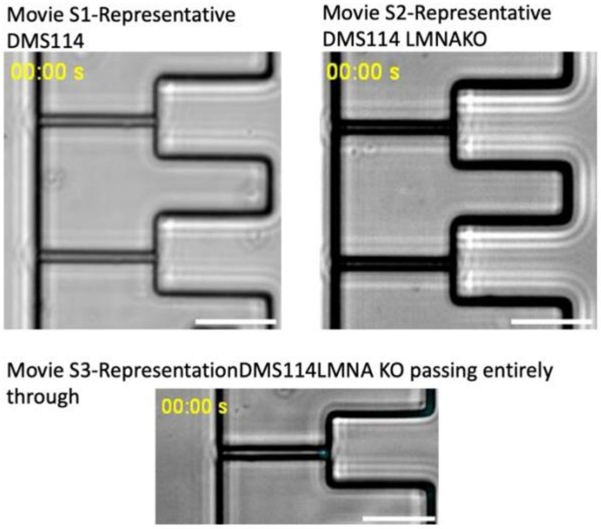

1. Febres-Aldana, C.A., et al., *Rb Tumor Suppressor in Small Cell Lung Cancer: Combined Genomic and IHC Analysis with a Description of a Distinct Rb-Proficient Subset.* Clin Cancer Res, 2022. **28**(21): p. 4702-4713.
2. Chan, J.M., et al., *Signatures of plasticity, metastasis, and immunosuppression in an atlas of human small cell lung cancer.* Cancer Cell, 2021. **39**(11): p. 1479-1496.e18.
