# Supplementary material for: Metastatic organotropism in small cell lung cancer
Source: bioRxiv. 2024 Oct 8:2024.10.07.617066. Preprint. [Version 1] doi: 10.1101/2024.10.07.617066 (PMC11483079; doi:10.1101/2024.10.07.617066)

Metastatic organotropism in small cell lung cancer

Supplementary Data

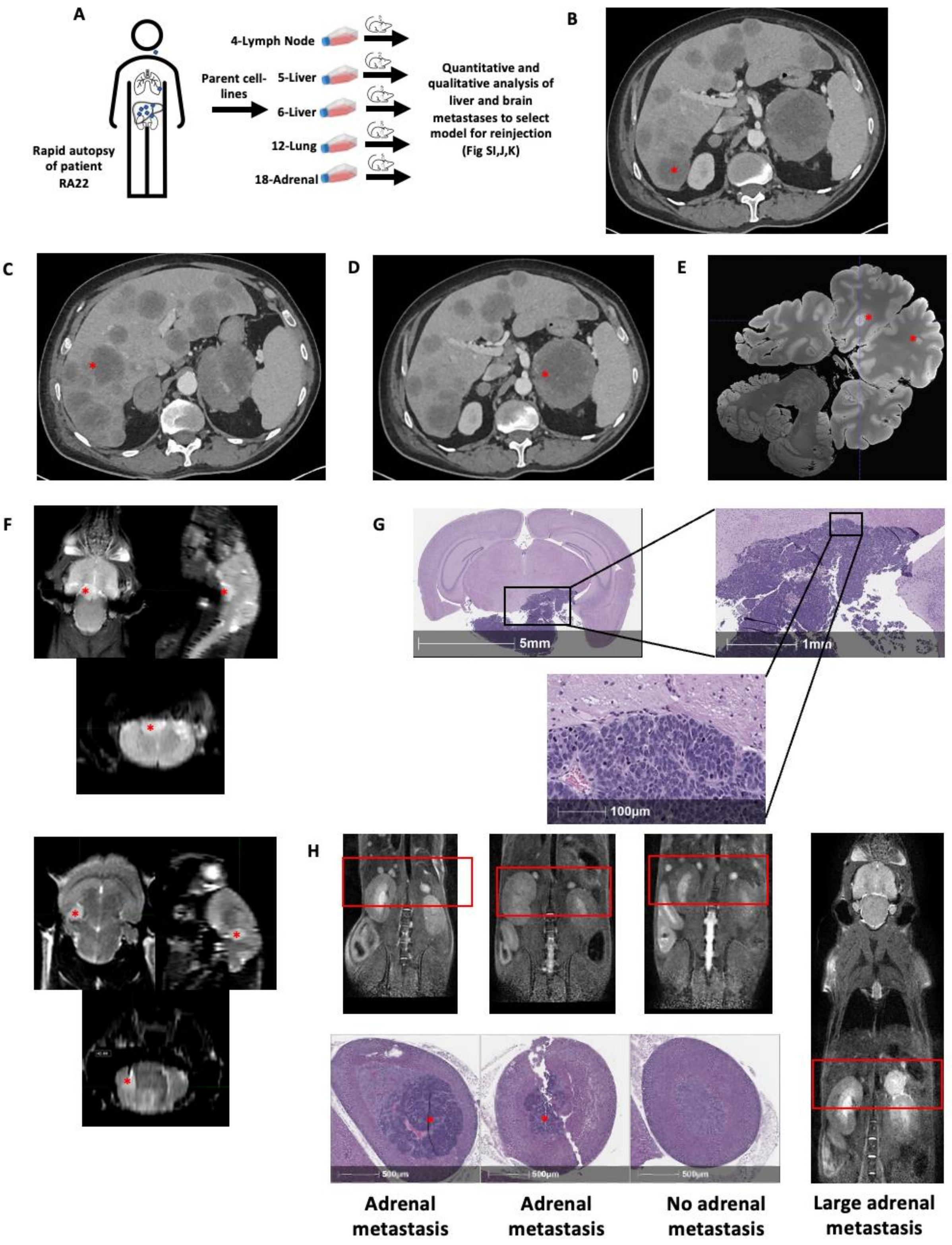

A
Rapid autopsy of patient RA22
Parent cell-lines
4-Lymph Node
5-Liver
6-Liver
12-Lung
18-Adrenal
Quantitative and qualitative analysis of liver and brain metastases to select model for reinjection (Fig SI,J,K)
B
C
D
E
F
G
5mm
1mm
100µm
H
500µm
500µm
500µm
Adrenal metastasis
Adrenal metastasis
No adrenal metastasis
Large adrenal metastasis

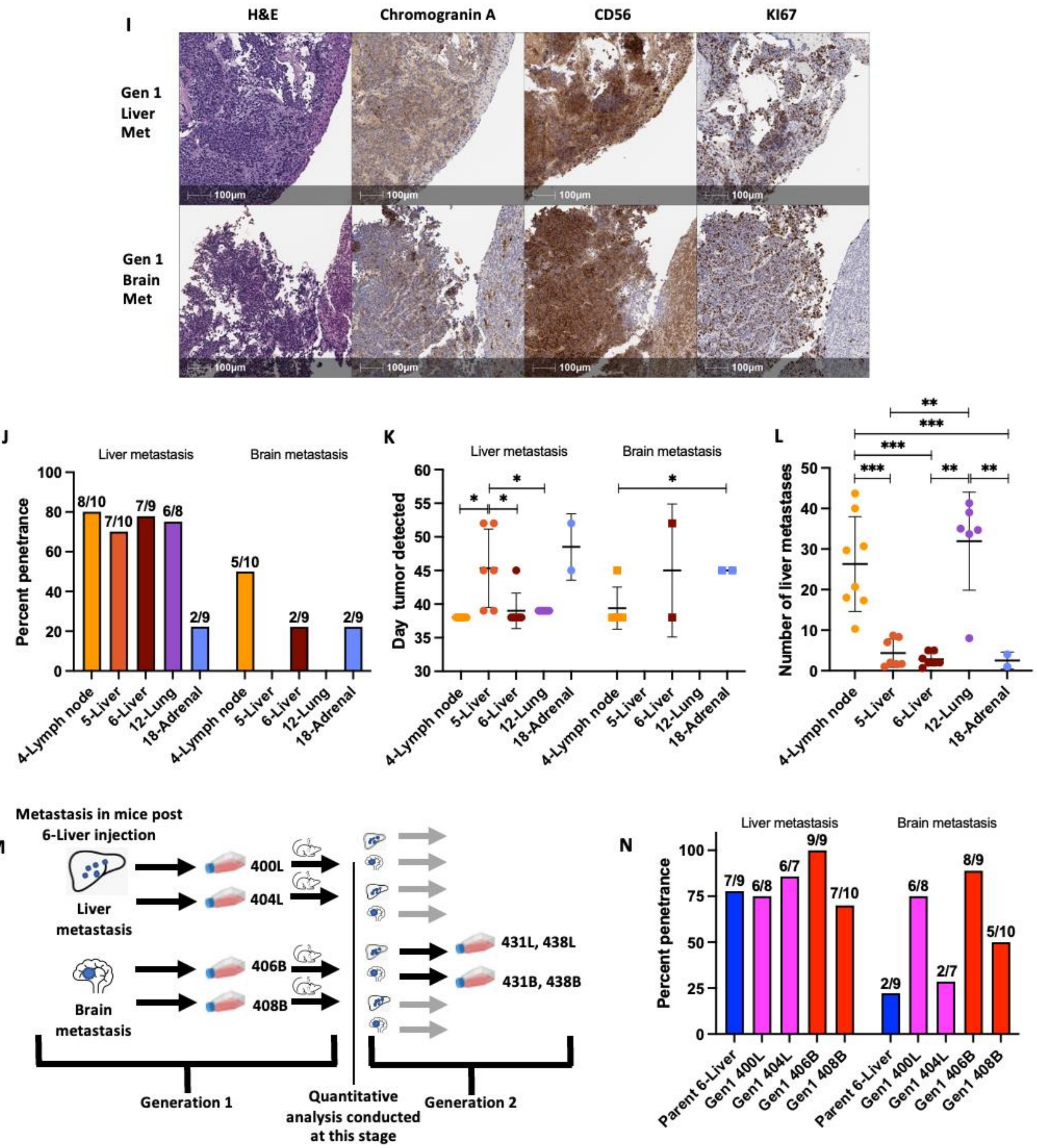

I
H&E
Chromogranin A
CD56
KI67
Gen 1 Liver Met
Gen 1 Brain Met
100µm
J
Liver metastasis
Brain metastasis
Percent penetrance
8/10
7/10
7/9
6/8
2/9
5/10
2/9
2/9
4-Lymph node
5-Liver
6-Liver
12-Lung
18-Adrenal
K
Liver metastasis
Brain metastasis
Day tumor detected
L
Number of liver metastases
M
Metastasis in mice post 6-Liver injection
Liver metastasis
Brain metastasis
400L
404L
406B
408B
431L, 438L
431B, 438B
Generation 1
Quantitative analysis conducted at this stage
Generation 2
N
Liver metastasis
Brain metastasis
Percent penetrance
7/9
6/8
6/7
9/9
7/10
2/9
6/8
2/7
8/9
5/10
Parent 6-Liver
Gen1 400L
Gen1 404L
Gen1 406B
Gen1 408B

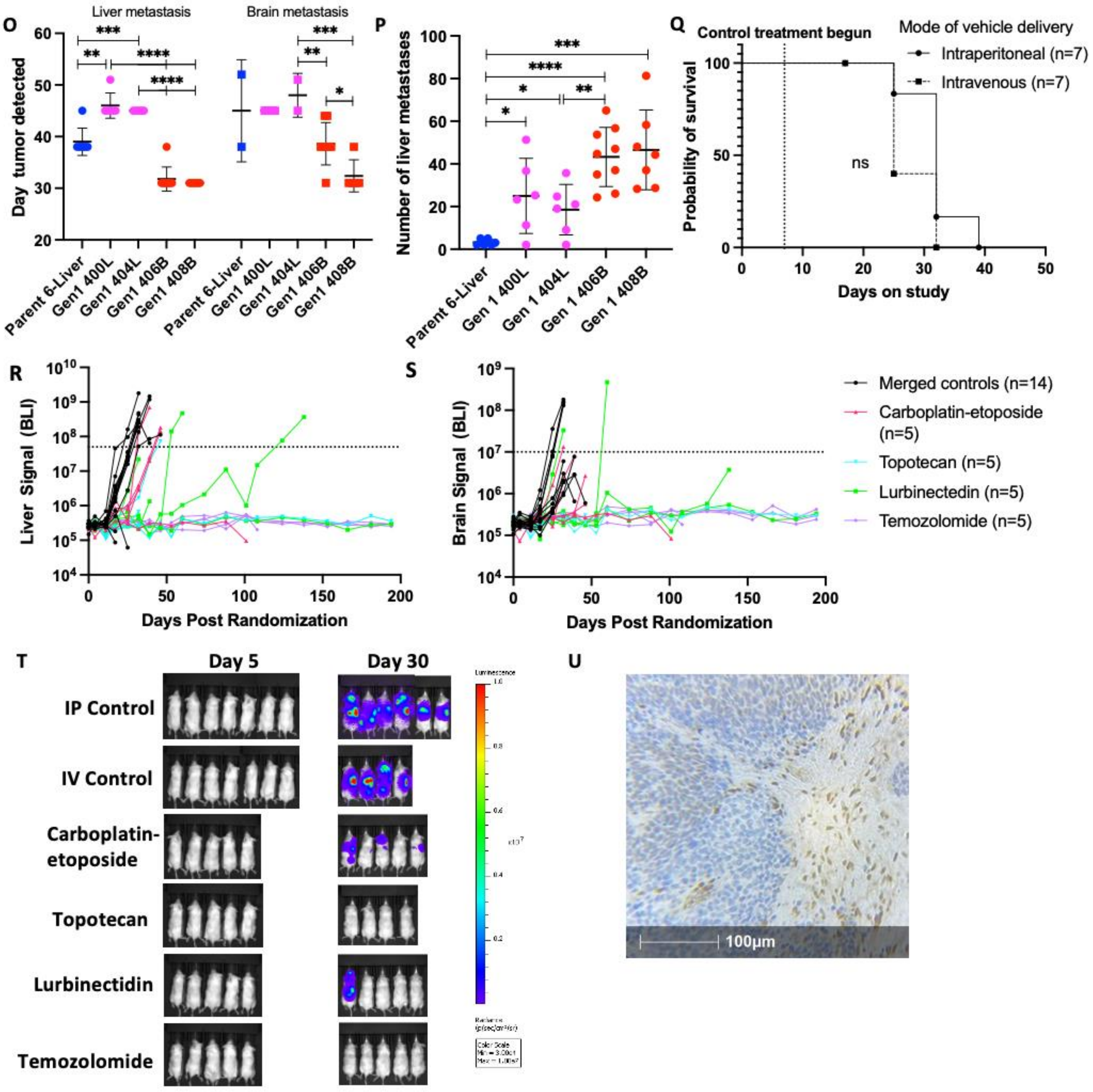

Figure S1: Establishment and characterization of a patient-derived model of SCLC metastases and organ tropism
A: Schema of mouse experiments with five parental cell lines.
B: CT scan for patient RA22 showing right posterior inferior lobe liver metastasis used to make 5-liver cell line, asterisks indicate metastasis
C: CT scan for patient RA22 showing right most inferior lobe liver metastasis used to make 6-liver cell line, asterisks indicate metastasis
D: CT scan for patient RA22 showing left adrenal metastasis used to make 18-adrenal cell line, asterisks indicate metastasis
E: Brain, CT post-mortem, asterisks indicate metastasis
F: Coronal, sagittal, and transverse cuts of typical (top) and rare (bottom) route of brain colonization, MRI, after RA22-4 (lymph node derived) cell line injection
G: Zoom of brain metastasis cells in Fig. 1F
H: Complexity identifying adrenal metastases on MRI
I: H&E and IHC for lung specific, tumor specific, and additional neuroendocrine factors in generation 1 metastases.

J: Percent penetrance (% of mice that formed tumors) of brain and liver metastases, assessed by MRI. RA22-4, RA22-5, RA22-6, RA22-12, RA22-18. Mice excluded if found dead or sacked due to bodyweight loss prior to appearance of first met in cohort. Mice numbers indicated above each bar.
K: Time to develop liver and brain metastasis in days. Mice with no metastases excluded.
L: Number of liver metastases. Mice with no metastases excluded
M: Schema of mouse experiments with five parental cell lines. RNA-seq data collected for generation 1 and 2 cell lines labeled.
N: Percent penetrance (% of mice that formed tumors) of brain and liver metastases, assessed by MRI. RA22-6, 400L, 404L, 406B, 408B. Mice excluded if found dead or sacked due to bodyweight loss prior to appearance of first met in cohort. Mice numbers indicated above each bar.
O: Time to develop liver and brain metastasis in days. Mice with no metastases excluded.
P: Number of liver metastases. Mice with no metastases excluded
Q: Survival (Kaplan-Meyer) curve of mice injected with 406B cells + vehicle. Vehicle delivered by routes indicated.
R: BLI signal from liver. The dotted horizontal line is the threshold for high tumor burden.
S: BLI signal from brain. The dotted horizontal line is the threshold for high tumor burden.
T: Representative image of BLI for each treatment condition
U: MGMT IHC from patient RA22 right most inferior lobe liver metastasis (used to make 6-liver cell line)

All significance indicated is following parametric unpaired T-tests with Welch's correction. *p<0.05, **p<0.01, ***p<0.001, ****p<0.0001. All error bars are mean with standard deviation unless mentioned otherwise. BMD: Brain metastasis derived; LMD cell lines: 400L, 404L; BMD cell lines: 406B, 408B. All patient Computational Topography (CT) transverse slices 2.5 weeks pre-mortem unless otherwise indicated.

Table S1

**Cell line**	**Procurement method**	**Site of metastasis**	**Generation**	**Cell line injected to yield**	**Generation + site name used throughout paper**
4-lymph node	Human rapid autopsy	Cervical lymph node	Parental		
5-liver	Human rapid autopsy	Right lobe posterior inferior liver	Parental		
6-liver	Human rapid autopsy	Right lobe inferior most	Parental		Parent
12-lung	Human rapid autopsy		Parental		
18-adrenal	Human rapid autopsy	Left adrenal	Parental		
400L	Mouse necropsy	Liver	1	6-liver	Gen1LMD
404L	Mouse necropsy	Liver	1	6-liver	Gen1LMD
406B	Mouse necropsy	Brain	1	6-liver	Gen1BMD
408B	Mouse necropsy	Brain	1	6-liver	Gen1BMD
431L	Mouse necropsy	Liver	2	406B	Gen2LMD
431B	Mouse necropsy	Brain	2	406B	Gen2BMD
438L	Mouse necropsy	Liver	2	406B	Gen2LMD

438B	Mouse necropsy	Brain	2	406B	Gen2BMD

Table S2:

Tumor Sample	Target	Marker Type	Typical [72]	IHC Type	Total Cells	% Positive	H-Score
RA-22-6	Chromogranin A	Traditional NE Marker	(74% cases)	Cytoplasmic	9111	89.5	210.3
RA-22-6	Synaptophysin	Traditional NE Marker	some not all cells	Cytoplasmic	15830	99.1	293.4
RA-22-6	INSM1	NE Marker	92%	Nuclear	74025	87.9	144.4
RA-22-6	CD56	Traditional NE Marker	75-100%(>80% of cases)	Cytoplasmic	27528	99.5	298.0
RA-22-6	Ki-67	Cancer Proliferative activity	60%	Nuclear	25155	58.7	93.2
406-Liver	Chromogranin A	Traditional NE Marker	(74% cases)	Cytoplasmic	5472	81.5	149.4
406-Liver	Synaptophysin	Traditional NE Marker	some not all cells	Cytoplasmic	3033	99.2	296.5
406-Liver	INSM1	NE Marker	92%	Nuclear	3671	74.3	87.6
406-Liver	CD56	Traditional NE Marker	75-100%(>80% of cases)	Cytoplasmic	2201	99.9	299.4
406-Liver	Ki-67	Cancer Proliferative activity	60%	Nuclear	2495	42.4	52.5
406-Brain	Chromogranin A	Traditional NE Marker	(74% cases)	Cytoplasmic	1396	48.7	75.9
406-Brain	Synaptophysin	Traditional NE Marker	some not all cells	Cytoplasmic	1786	99.7	298.7
406-Brain	INSM1	NE Marker	92%	Nuclear	2079	89.2	124.1
406-Brain	CD56	Traditional NE Marker	75-100%(>80% of cases)	Cytoplasmic	1801	99.8	297.6
406-Brain	Ki-67	Cancer Proliferative activity	60%	Nuclear	17530	58.1	86.0

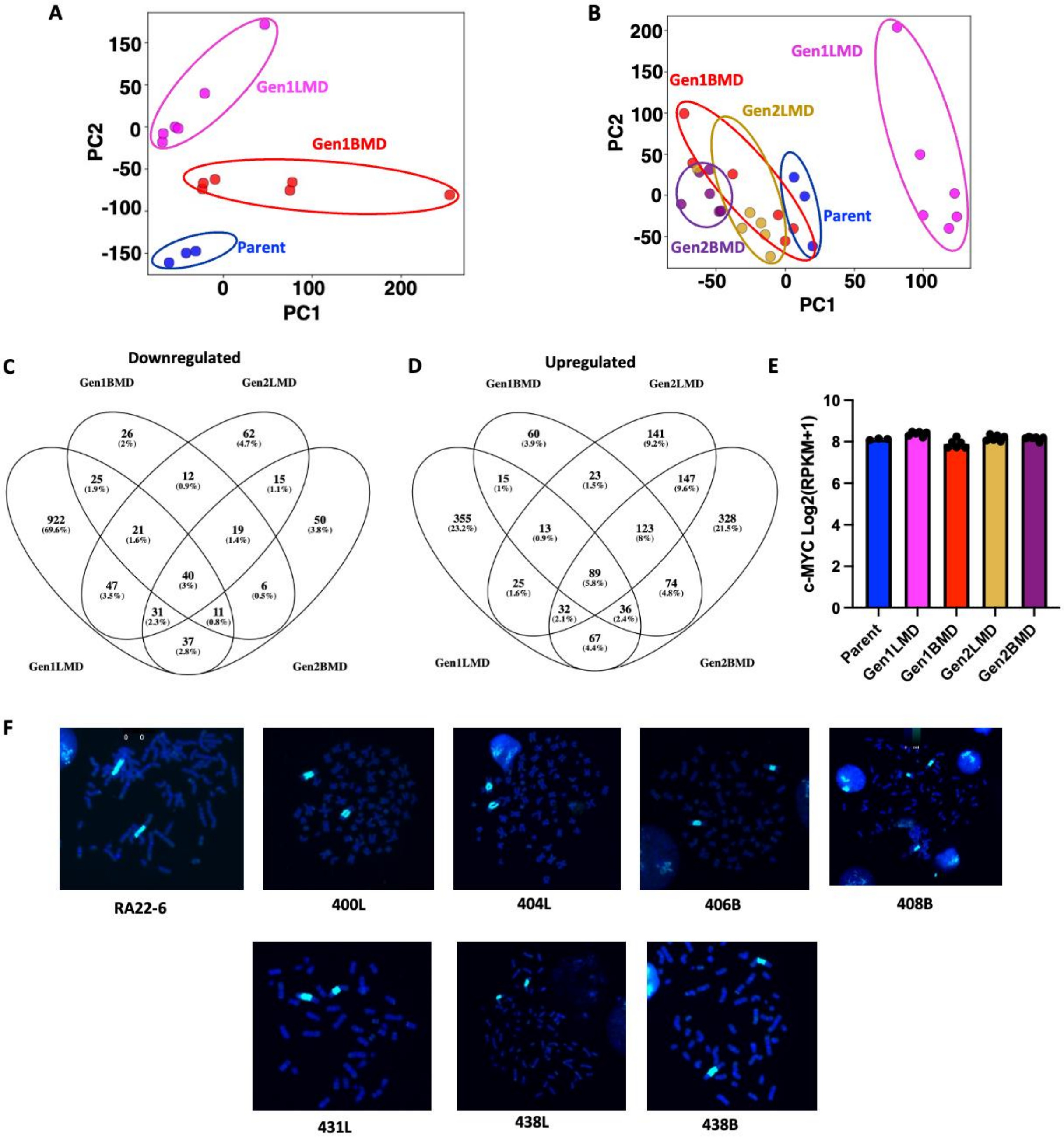

Figure S2: Global gene expression analysis of RA22-6 and derived cell lines

A: PCA of Parent and generation 1 all genes RNA-seq

B: PCA of Parent, generation 1, and generation 2 all genes RNA-seq

C: Venn diagram depicting differentially (Pvalue <0.05 & abs(logFC) >1.5) downregulated genes in Gen1LMD, Gen2LMD, Gen1BMD, Gen2BMD compared to Parent.

D: Venn diagram depicting differentially (Pvalue <0.05 & abs(logFC) >1.5) upregulated genes in Gen1LMD, Gen2LMD, Gen1BMD, Gen2BMD compared to Parent.

E: Gene expression of MYC

F: MYC DNA-FISH for MYC in Parent RA22-6, generation 1, and generation 2 cell lines

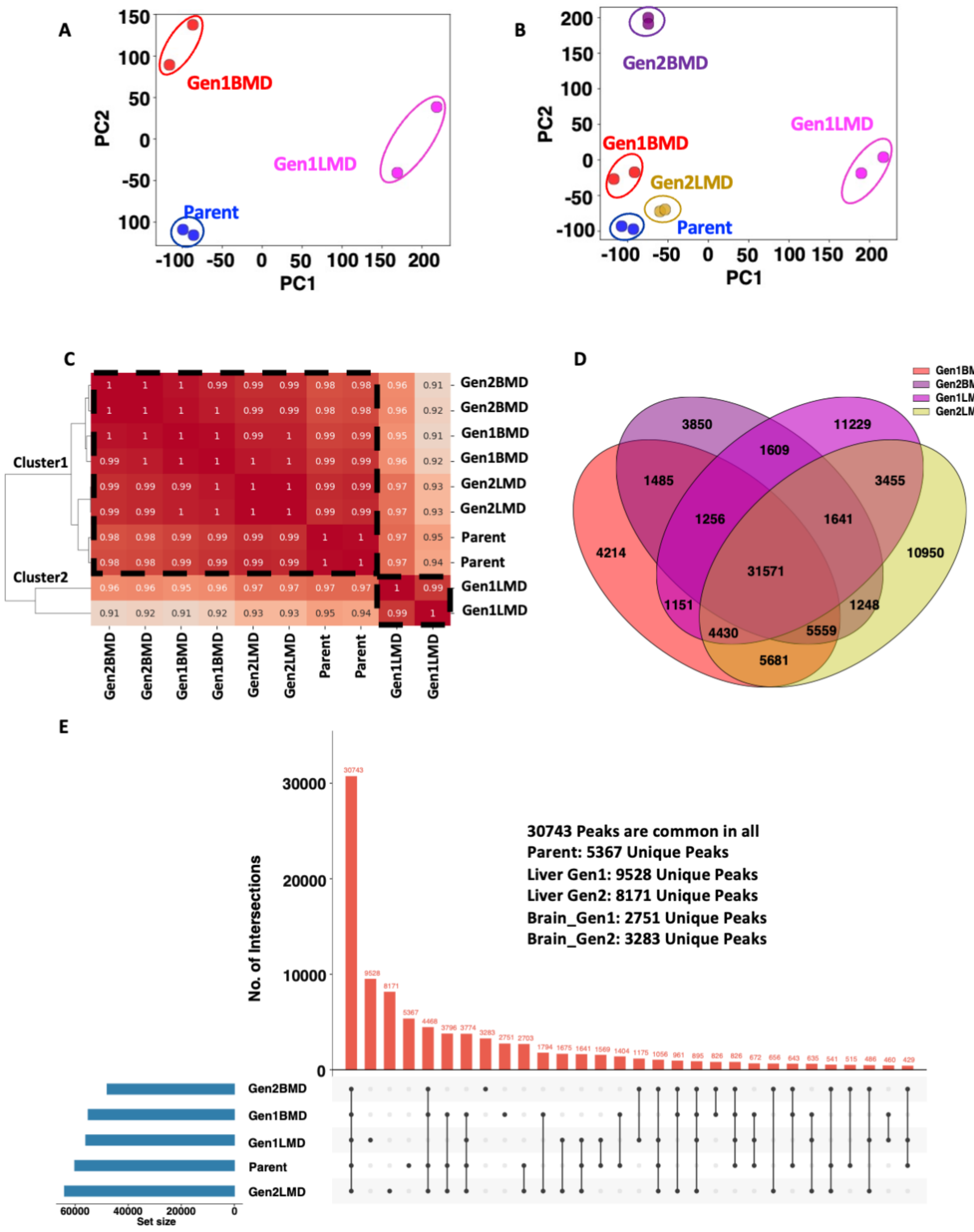

Figure S3: Chromatin accessibility associated with SCLC organ-specific metastatic behaviors

A: PCA of Parent and generation 1 all peaks ATAC-seq

B: PCA of Parent, generation 1, and generation 2 all peaks ATAC-seq
C: Pairwise correlation heatmap of open peaks in Parent, generation 1, and generation 2.
D: Venn Diagram depicting unique peaks between Gen1LMD, Gen1BMD, Gen2LMD and Gen2BMD.
E: Bar graph depicting overlap of peaks between Parent, generation 1, and generation 2 all peaks

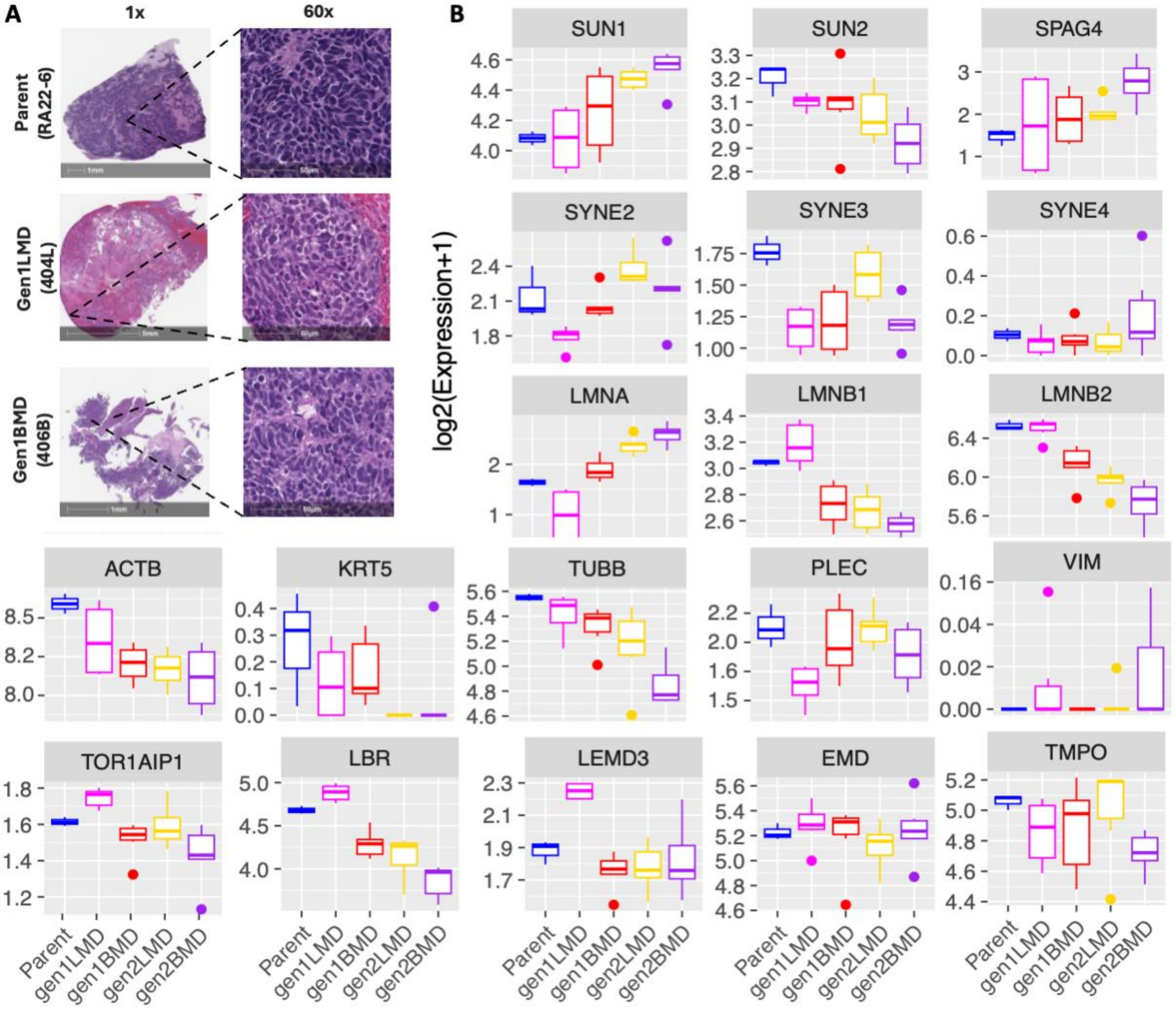
A
1x
60x
Parent (RA22-6)
Gen1LMD (404L)
Gen1BMD (406B)
B
log2(Expression+1)
SUN1
SUN2
SPAG4
SYNE2
SYNE3
SYNE4
LMNA
LMNB1
LMNB2
ACTB
KRT5
TUBB
PLEC
VIM
TOR1AIP1
LBR
LEMD3
EMD
TMPO
Parent
gen1LMD
gen1BMD
gen2LMD
gen2BMD

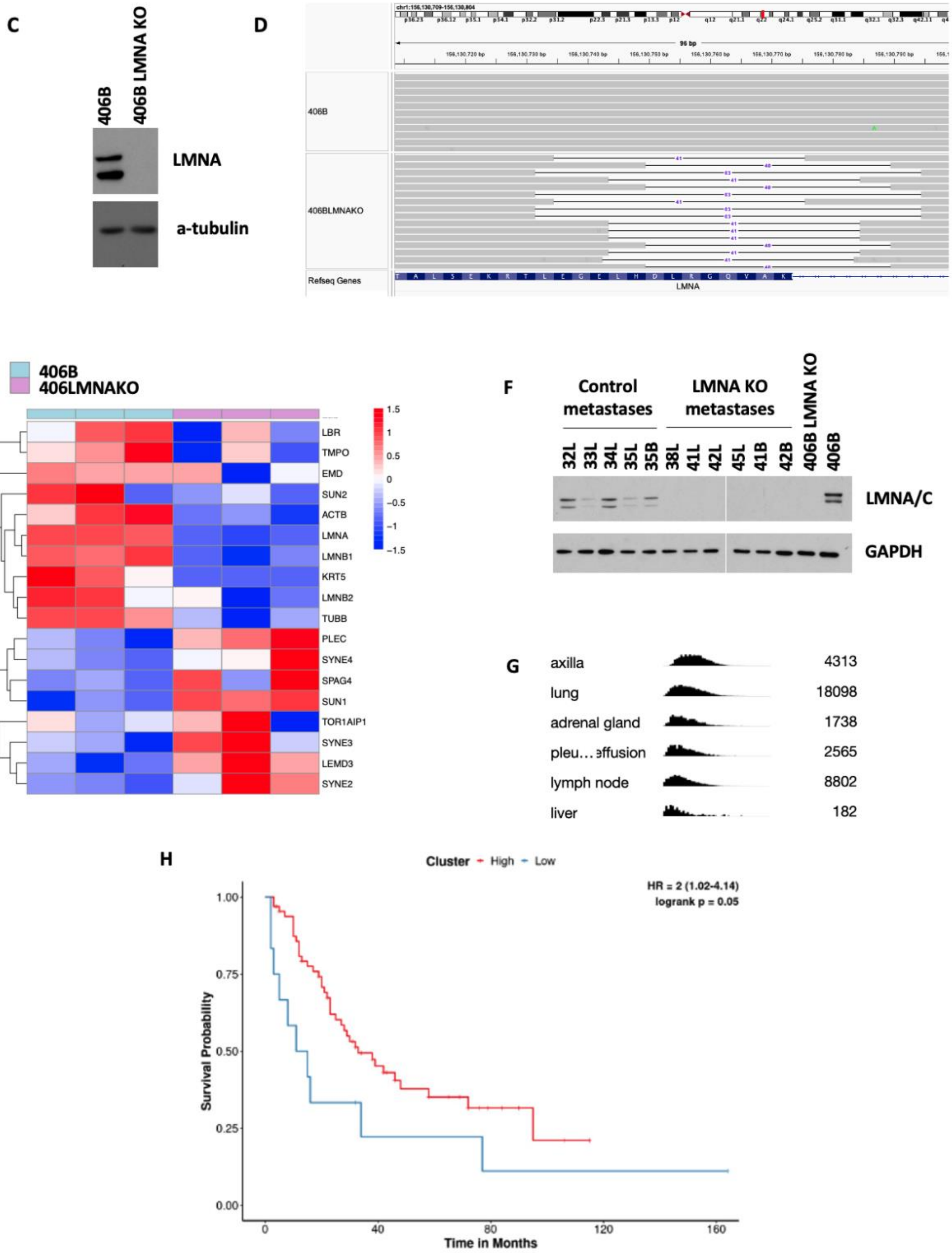

Figure S4: Dysregulation of cellular structure and LMNA KO in SCLC

A: H&E illustrating SCLC nuclei in Parent and generation 1 metastases

B: RNA-seq of LINC complex genes in Parent, generation 1, and generation 2 of SCLC metastasis model (shows progressive dysregulation of LINC)
C: Western blot of LMNA KO in 406B
D: Deep amplicon sequencing to identify mutations in 406B LMNAKO
E: Heatmap of gene expression of LINC complex and related genes in 406B and 406B LMNAKO
F: LMNA/C western blot from metastasis cell lines after in vivo LMNAKO metastasis modeling. Samples were run side-by-side on two different blots (separated by white space above) that were developed at the same time.
G: Distribution of LMNA expression of human SCLC metastatic cells [46] expressing, LMNA stratified by site of metastasis
H: LMNA expression and survival (n=77 patients) [50], stratified lower 15th percentile of LMNA expression compared to the rest.

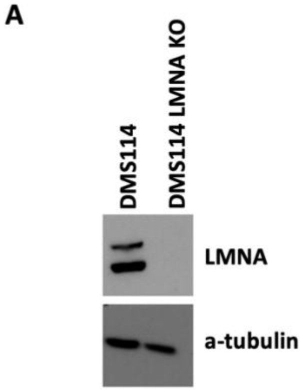

Figure S5: In vitro examination of LMNA role in nuclear deformability, migration, and metastasis
A: Western blot of LMNA KO in DMS114

Movies:

Movie S1-Representative DMS114

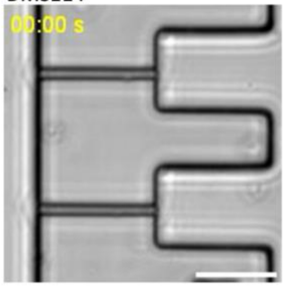

Movie S2-Representative DMS114 LMNAKO

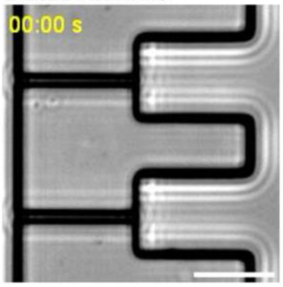

Movie S3-RepresentationDMS114LMNA KO passing entirely through

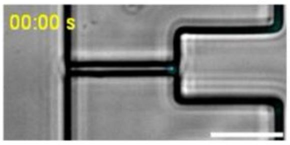

Supplement: Supplementary file 1 [file media-1.pdf]
